# Unicuspid Aortic Valve Disease: The Role of Imaging in Diagnosis and Timely Surgical Planning

**DOI:** 10.7759/cureus.93084

**Published:** 2025-09-24

**Authors:** Mehak Gupta, Aaditya Kodamanchile, Marina Zafeiri, Pavithralakshmi Venkatraghavan, Andrew Cole

**Affiliations:** 1 Department of Cardiology, University Hospitals of North Midlands NHS Trust, Stoke-on-Trent, GBR; 2 Department of Medicine, University Hospital Southampton NHS Foundation Trust, Southampton, GBR; 3 Department of Medicine, Hereford County Hospital, Hereford, GBR

**Keywords:** aortic valve replacement, cardiac mri, transesophageal echocardiogram, transthoracic echocardiogram, unicuspid aortic valve

## Abstract

Unicuspid aortic valve (UAV) is a rare congenital anomaly that is often misdiagnosed as bicuspid aortic valve (BAV). UAV is associated with faster clinical progression, earlier need for surgical intervention, and a higher risk of complications. Accurate diagnosis is therefore crucial for timely management. We report the case of a 31-year-old male with a family history of BAV, who had been followed for more than 15 years with a presumed diagnosis of BAV. Serial transthoracic echocardiograms (TTEs) and cardiac MRI consistently suggested a bicuspid morphology. A previous transesophageal echocardiogram (TOE) was poorly tolerated. Despite progressive aortic regurgitation and root dilation, the diagnosis remained unchanged until the patient developed exertional dyspnea and presyncope. A repeat TOE revealed a unicuspid valve with severe eccentric regurgitation, which was later confirmed intraoperatively during aortic valve replacement with concomitant ascending aortic surgery. This case highlights the diagnostic challenges of UAV, the limitations of TTE and MRI in assessing valve morphology, and the pivotal role of TOE. Early clinical suspicion and timely multimodal imaging are essential to avoid delayed recognition and to optimize surgical planning and outcomes.

## Introduction

Unicuspid aortic valve (UAV) is a rare congenital anomaly, with an estimated prevalence of 0.02-0.06% [1,2]. UAV may be either unicommissural or acommissural and is frequently misclassified as a bicuspid aortic valve (BAV) because of overlapping echocardiographic features [3]. Unlike BAV, UAV progresses more rapidly, and patients often require aortic valve replacement (AVR) one to two decades earlier [4].

Here, we present the case of a patient with a UAV that was misdiagnosed as BAV for more than 15 years, highlighting the importance of multimodal imaging and timely diagnosis.

## Case presentation

A 31-year-old male with a family history of BAV and aortic regurgitation (AR) (brother diagnosed at age 13) was referred to cardiology services in 2007. His initial transthoracic echocardiogram (TTE) revealed mild AR, a left ventricular ejection fraction (LVEF) of 45%, and raised suspicion of BAV, although valve morphology could not be clearly visualized. Following this, he underwent yearly serial TTEs. From 2007 through 2020, there were no significant changes in his echocardiographic findings, and clinically, he remained asymptomatic.

In 2020, his TTE showed progression of AR into the moderate range, with a mild increase in left ventricular (LV) cavity size. He subsequently underwent a transesophageal echocardiogram (TOE), which he did not tolerate well. The suboptimal images obtained appeared to show moderate AR with a dilated LV and moderately impaired systolic function (LVEF 45%). His aortic dimensions were normal for his body surface area. As he remained clinically asymptomatic at this point, he continued with yearly TTE surveillance.

**Figure 1:**
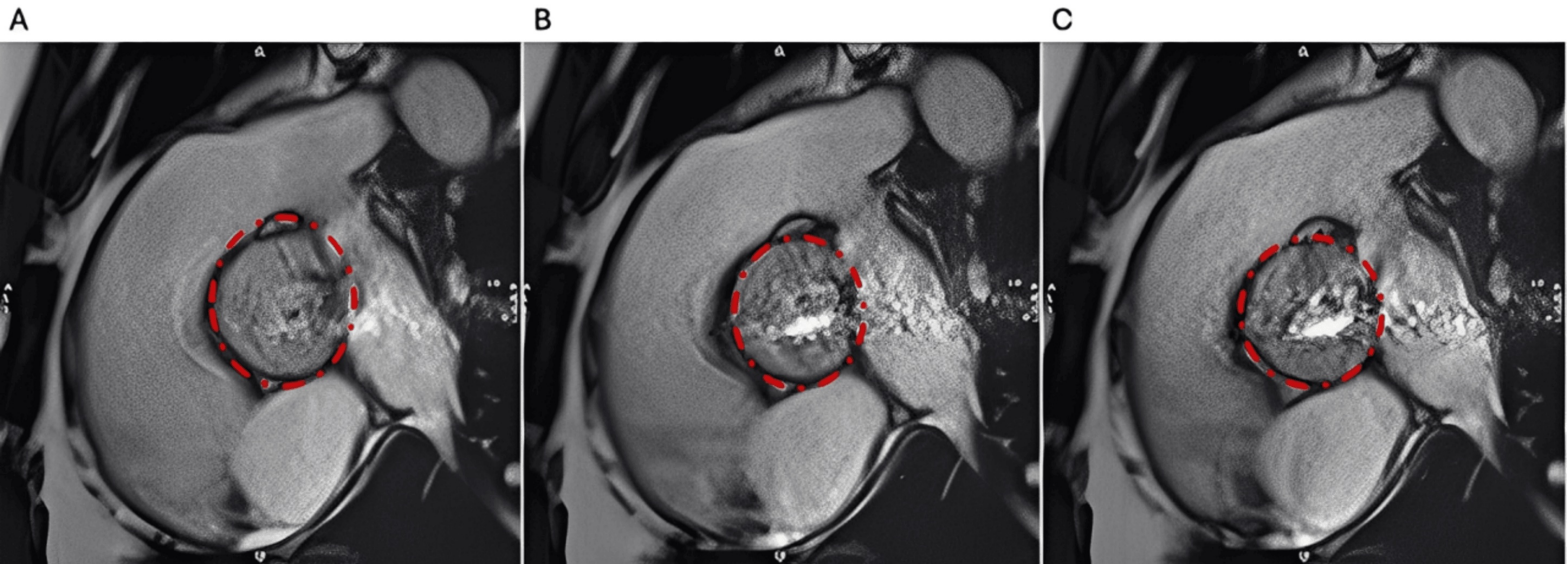
Cardiac MRI still frames of a UAV obtained with SSFP cine sequences during different phases of the cardiac cycle: (A) diastole, (B) mid-systole, and (C) end-systole The red dashed circle indicates the aortic valve. SSFP, steady-state free precession; UAV, unicuspid aortic valve

In 2024, he developed exertional breathlessness and dizziness. A repeat TTE, which did not adequately visualize the aorta, showed a significantly dilated LV with an LVEF of 48% and at least moderate AR. His AR appeared complex, with two distinct jets, raising concern that its severity was being underestimated. To further characterize his aortic dimensions, he underwent CT cardiac angiography, which revealed a dilated aortic root measuring 42 × 30 mm and an ascending aorta measuring 43 mm, with a BAV. Cardiac MRI subsequently demonstrated a moderately dilated LV (LVEF 54%), with the aortic valve again reported as bicuspid morphology with severe AR (Figure 1).

**Figure 2:**
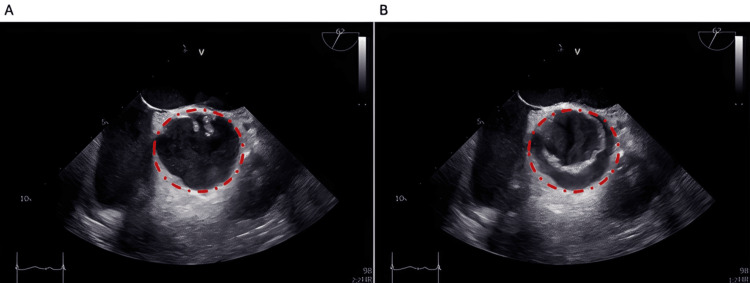
Transesophageal echocardiographic images of a UAV in (A) diastole and (B) systole, obtained in the short-axis aortic view The red dashed circle indicates the aortic valve. UAV, unicuspid aortic valve

He later underwent a repeat TOE, which showed a mildly stenotic UAV with severe eccentric regurgitation and a dilated LV (LVEF 51%) (Figure 2). His case was discussed at a valve multidisciplinary team (MDT) meeting. On re-review of his previous imaging, the MRI was considered to show a UAV. The MDT recommended surgical intervention with replacement of the aortic valve, aortic root, and ascending aorta.

He underwent a modified Bentall procedure with replacement of the ascending aorta using a 27 mm On-X composite Valsalva graft. The operation was carried out routinely, with a total cross-clamp time of 334 minutes and a bypass time of 385 minutes. Intraoperative inspection of the native aortic valve confirmed a UAV.

## Discussion

This case highlights the limitations of TTE and cardiac MRI in diagnosing UAV, particularly when overlapping features with BAV are present. TTE is widely used as the first-line imaging modality to assess valvular pathology because of its availability and tolerability. However, it is often inadequate as a sole technique due to restricted acoustic windows. Cardiac MRI cine imaging is generally robust for functional and morphological assessment, but in this case, the interpretation of the aortic valve was likely influenced by anchoring bias from the long-standing assumption and repeated reports of BAV, resulting in misclassification despite adequate image quality [5,6].

In certain cases, TOE, with its superior resolution and proximity to the aortic valve, provides additional information for assessing valve morphology and the fusion pattern characteristic of UAV [7]. However, because TOE is more invasive, patients often find it difficult to tolerate.

In this case, delayed recognition of the UAV may have influenced the intensity of surveillance and the timing of intervention. More importantly, it illustrates the effect of anchoring bias, where the initial assumption of BAV, reinforced by repeated TTE and MRI reports, led clinicians to discount alternative diagnoses despite progressive changes. Awareness of such cognitive bias is critical in rare congenital anomalies to prevent diagnostic delays.

Clinically, UAV has implications beyond valve replacement, as it carries a higher risk of root dilatation and the need for concomitant aortic surgery (Table 1). UAV is associated with early development of aortic root dilation and AR and therefore predominantly affects younger patients, in whom aortic surgery is the main consideration. A subset of patients may not be suitable candidates for surgery; although the role of transcatheter AVR remains limited at present, it may emerge as a treatment option for frailer, high-risk surgical candidates [8].

**Table 1:** Imaging and clinical characteristics differentiating UAV from BAV Data adapted from [1,3-5,7,8]. AR, aortic regurgitation; BAV, bicuspid aortic valve; TOE, transesophageal echocardiogram; TTE, transthoracic echocardiogram; UAV, unicuspid aortic valve

Feature	UAV	BAV
Prevalence	0.02-0.06% (rare)	1-2% (more common)
Valve morphology	Single commissure (unicommissural) or none (acommissural)	Two commissures with asymmetric leaflet fusion
Diagnostic challenge	Often misdiagnosed as BAV on TTE/MRI	Usually identifiable, though variants exist
Progression	More rapid, earlier degeneration	Slower progression
Age at surgery	20-30 years earlier than tricuspid; ~10-20 years earlier than BAV	Typically, middle-aged or older adults
Common pathology	Aortic stenosis (most frequent), severe AR, root dilatation	Aortic stenosis with/without regurgitation; root dilatation
Risk of complications	High risk of dissection, aneurysm, and root enlargement	Elevated risk, but generally later onset
Best imaging modality	TOE (superior resolution for commissures)	TTE sufficient in most cases

UAV and BAV share a common genetic predisposition, but UAV appears to represent a more severe phenotype, with earlier onset and faster progression of aortic stenosis. One study comparing UAV and BAV demonstrated that patients with UAV had smaller indexed aortic valve areas and higher mean and peak gradients than those with BAV. This supports the concept of UAV as a more aggressive phenotype within the BAV spectrum, presenting with more severe pathology at a younger age [9]. Given the family history in this case, genetic predisposition should be considered, and early, detailed imaging of first-degree relatives may be warranted.

## Conclusions

We report a rare case of a UAV misdiagnosed as BAV for more than 15 years, ultimately identified by TOE and confirmed intraoperatively. We suspect that this prolonged misdiagnosis was influenced by anchoring bias. This case underscores the importance of employing multimodality imaging in the evaluation of valvular pathology and highlights the need for independent image review to minimize reliance on prior reports.
